# Pediatric Cholestatic Diseases in the Era of Ileal Bile Acid Transporter (IBAT) Inhibitors

**DOI:** 10.3390/pediatric18010019

**Published:** 2026-02-03

**Authors:** Marco Sciveres, Silvio Veraldi, Francesco Cirillo, Giuseppe Maggiore

**Affiliations:** 1 Hepatology and Liver Transplantation, IRCCS Bambino Gesù Paediatric Hospital, 00165 Rome, Italy; silvio.veraldi@opbg.net (S.V.);; 2 Pediatric Gastroenterology and Hepatology Unit, Santobono-Pausillipon Children’s Hospital, 80129 Naples, Italy; f.cirillo@santobonopausilipon.it

**Keywords:** pediatric cholestasis diseases, PFIC, Alagille syndrome, cholestatic pruritus, IBAT inhibitor

## Abstract

Cholestatic diseases in children represent a heterogeneous group of disorders that, with few exceptions, have no cure. For decades, off-label drugs and/or drugs with little evidence of efficacy have been used to treat pruritus or as supportive therapy. In recent years, a family of molecules known as bile acid transporter inhibitors (IBATis) has been developed, with two of these being approved for treating pruritus in progressive familial intrahepatic cholestasis (PFIC) and Alagille syndrome (ALGS). Blocking the ileal reabsorption of bile acids (BAs) lowers serum levels. This contributes to reducing cholestatic pruritus. Such a mechanism of action may also have a potential benefit in other cholestatic diseases and even in the consequences of chronic cholestasis. This is a narrative review of the literature, including the most recent communications, to summarize data on the efficacy and safety of IBATis in the treatment of pruritus in PFIC and ALGS in children, including a description of the latest results from their use in a real-world setting. Reports on off-label use and experiences in adults are also discussed. This review aims to help physicians understand the potential and limitations of these new drugs in the treatment of cholestatic pruritus.

## 1. Introduction and Aims

Hundreds of proteins contribute to the biosynthesis and excretion of bile components from hepatocytes to the biliary tree. Mutations in each of the genes encoding these proteins may be the cause of an inherited monogenic disease. In addition, a heterogeneous group of acquired cholestatic disorders exist, including biliary atresia, which is the most prevalent cholestatic disease in infants [1].

Although rare individually, taken together, cholestatic diseases represent the commonest indication for liver transplantation (LT) in any age group, accounting for between 50 and 60% of all pediatric liver transplants [2]. Each year, approximately 300 children in Europe and the United States undergo LT for cholestatic diseases [3].

A real cure is exceptionally available, such as cholic acid in bile acid synthesis defects. The prognosis of the majority of diseases depends essentially on the severity of the phenotype. Most affected children will require a liver transplant at some point.

Cholestasis has several clinical consequences, including malabsorption of lipids and fat-soluble vitamins, retention of bile components in the blood and progressive liver damage secondary to cell death and fibrosis [4]. Itching may be the most disabling symptom of cholestasis. The cause of pruritus is not fully understood. Several molecules probably play a role. Among them, bile acids are the most known. In most cases, the serum concentration of BA reflects the severity of itching, albeit imperfectly [5].

Occasionally intractable itching is the main indication for transplantation, even in the presence of a liver with sufficient functional reserve [4]. Treatment of pruritus, however, has been unsatisfactory so far. Until recently off-label drugs and/or drugs with little evidence of efficacy have been used to treat pruritus. In recent years a new class of drugs, called inhibitors of ileal bile acid transporters, has successfully completed randomized controlled trials (RCTs). They are currently approved both in Europe than in the US for the treatment of pruritus in PFIC and ALGS.

The aim of this review is to provide a summary of the existing evidence on the effectiveness of IBATis in treating cholestatic pruritus in children. We have considered both approved indications and both available molecules to provide a broader overview. Moreover, special attention was paid to real-world data and off-label use in order to better assess the impact of this new class of drugs in daily practice. The main limitation of this work is that recent data from real-world case series are only available in the form of non-peer-reviewed abstracts or in the context of small case series within multicenter observational studies.

## 2. Methods

This is a narrative review. However, a structured literature search was carried out using the electronic database PubMed with no time restrictions in October 2025. The search terms used were as follows: (maralixibat) OR (odevixibat) OR volixibat OR (elobixibat) OR (linerixibat). A total of 82 papers were considered, excluding reviews. However, we also decided to extend the research to include abstracts presented at major international conferences, as the most recent real-world study results have not yet been fully published. Additionally, some secondary outcomes from registration studies were made public after the main results were published and are currently only available in abstract form. For this reason, we reviewed the proceedings from the annual meetings of the AASLD, EASL and ESPGHAN over the last three years (2023–2025).

We only considered abstracts that report on the sub-analysis results of registration studies or results from open-label extension studies or issued from collaborations within national scientific societies. However, it should be noted that these data have not been peer-reviewed and should therefore be interpreted with caution. Citations that refer to congress proceedings are clearly marked in both the main text and the references section.

## 3. Cholestatic Diseases of Infancy and Childhood

A number of liver diseases may present with cholestatic features. Here, we summarize only the most relevant with regard to experience with IBATis.

**Progressive familial intrahepatic cholestasis** is a group of monogenic diseases with a marked cholestatic phenotype characterized by a defect in a protein involved in the process of bile formation and excretion [6]. A total of 13 variants have been recognized so far (Table 1) [7].

**PFIC1** is defined by a defect in the familial intrahepatic cholestasis 1 (FIC1) protein, encoded by the *ATPB1* gene. FIC1 is an aminophospholipid flippase that creates an imbalance in the distribution of membrane phospholipids by translocating phosphatidylserine to the inner side [8]. This imbalance is probably necessary for the functioning of several membrane pumps including the bile salt export pump (BSEP) or the cystic fibrosis transmembrane conductance regulator (CFTR). FIC1 expression is ubiquitous; affected patients have intrahepatic cholestasis and pruritus but usually also extrahepatic manifestations such as failure to thrive, chronic diarrhea and malabsorption [8].

**Figure 1:**
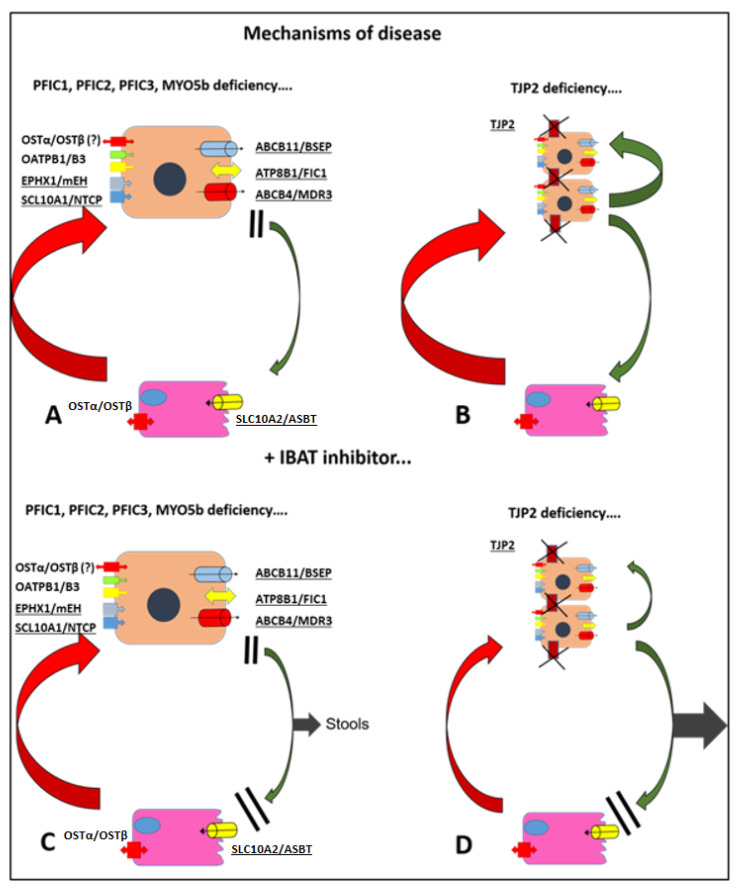
Mechanisms of disease in PFIC and consequences of IBATis therapy. (**A**) Mechanism of disease in PFIC 1-2-3-10. Defective membrane transporters impair secretion of bile acids in the bile. (**B**) Mechanism of disease in PFIC4. Membrane transporters are intact, but defective tight junctions allow bile acids to reflux in the intercellular compartment. (**C**) Administration of IBATis in PFIC 1-2-3-10 causes loss with stools of a relatively small amount of bile acids. (**D**) Administration of IBATis in PFIC4 results in a significant loss of bile acids and effective and quick reduction in the blood pool. **Abbreviations.** OSTα/β: organic solute transporter alpha/beta; OATPB1/3: organic anion transporting polypeptides B1/3; mEH: microsomal epoxide hydrolase; NTCP: sodium taurocholate co-transporting polypeptide; BSEP: bile salt export pump; FIC1: familial intrahepatic cholestasis type 1; MDR3: multidrug resistance protein 3; ASBT: apical sodium-dependent bile acid transporter.

BSEP defect characterizes **PFIC2**. This protein is the main transporter of BA in bile (Figure 1A). The bile of affected children has a very low or absent BA concentration while hepatocytes suffer severe and early damage due to the high intracellular concentration. It is an exclusively hepatic disease characterized by severe itching, high alpha-fetoprotein values and a pronounced tendency to carcinogenesis [9].

**PFIC3** is caused by mutations in the *ABCB4* gene, which encodes the multidrug resistance 3 (MDR3) protein. MDR3 is a floppase that enriches the outer leaflet of the canalicular membrane with phosphatidylcholine, enabling the extraction and incorporation of phosphatidylcholine into the biliary mixed micelles. Bile phospholipids are essential to prevent injury to biliary epithelia caused by hydrophobic BA. Patients produce toxic, very low phospholipids containing bile and typically have high GGT activity [10]. PFIC3 patients may also present in late childhood with chronic cholangitis and advanced fibrosis, mild itching and signs of portal hypertension [11].

**PFIC4** is caused by the malfunction of tight junction protein 2 (TJP2). Nonfunctional tight junctions allow the reflux of BA and other components of bile in the paracellular compartment, ultimately causing damage and fibrosis. Severity of disease is highly variable, and symptoms include jaundice, hepatomegaly, itching, usually low GGT and elevated serum bile acids (sBAs) at laboratory assessment [12]. The mechanisms of cholestasis are very different from PFIC 1-2-3 and are summarized in Figure 1B.

**PFIC10** is caused by defective intracellular trafficking, due to mutated myosin-Vb protein (MYO5B), resulting in incorrect positioning of canalicular transporters, including BSEP. Pathogenesis of cholestasis is similar to PFIC 2 with a very low biliary concentration of BA. Symptoms include jaundice and severe itching, but extrahepatic features are common such as failure to thrive and diarrhea. The same gene is causative of microvillous inclusion disease (MVID) with a complex genotype–phenotype correlation [13].

**Alagille syndrome (ALGS)** is an inherited, multiorgan disease caused in over 90% of cases by mutations of the *JAG1* gene or in 2–3% of cases by mutations in *NOTCH2*. It is inherited as an autosomal dominant disease but up to 60% of mutations are de novo. The Notch signaling pathway is critical for cell-to-cell communication during embryogenesis. Jagged 1 is one of the ligands of the four Notch receptors, and its correct expression in the liver is critical for the development of a normal intrahepatic biliary tree. Other affected organs include the heart and vascular system, kidney, bones and eyes, and patients have typical facial features. The hepatic phenotype of ALGS is most commonly neonatal cholestasis with high GGT. Pruritus is usually severe in early childhood, while older children tend to show signs of portal hypertension, though cholestasis is milder [14]. In ALGS, the pathogenesis of cholestasis is essentially obstructive, and interlobular biliary ducts are often absent or dysmorphic.

**Biliary atresia** is the most common cholestatic disease affecting 30–40% of cholestatic newborns. Early and progressive fibrosis and obliteration of both intra- and extrahepatic biliary ducts cause severe and irreversible cholestasis. It is an acquired disease; children in most cases are born healthy. This is the prototype of obstructive cholestasis; untreated children quickly develop terminal biliary cirrhosis and usually die by the age of two. Early Kasai portoenterostomy can restore a certain amount of biliary flow, but ultimately most children will need a liver transplant [15]. Itching is usually present in small children with decompensated cirrhosis waiting for liver transplant or in older children with bile flow only partially restored.

**Sclerosing cholangitis** in children is in most cases an acquired, autoimmune disease. Patients are typically young adolescents, and most of them suffer also from inflammatory bowel disease. Cholestasis occurs in advanced stages of the disease when fibrosclerosis of the bile ducts causes mechanical stenosis. However, retention of BA may contribute to the scarring process by promoting periductal fibrosis [16].

## 4. IBAT Inhibitors

After hepatic synthesis and intestinal excretion mainly through BSEP, BAs are actively reabsorbed into the final ileal loop by IBAT, a member of the solute carrier (SLC) family, encoded by the *SLC10A2* gene [17]. IBAT functions as a membrane-bound transporter that actively co-transports conjugated BAs using the drive of sodium gradients into enterocytes. From there, BAs cross the basolateral membrane via organic solute transporters (OST) α and β and reach the liver with the portal blood (Figure 1). The sodium taurocholate co-transporting polypeptide (NTCP) closes the enterohepatic cycle by uptaking BAs again into hepatocytes [18]. Intestinal reabsorption guarantees over 95% efficiency with only 5% of BAs being lost with stools. IBAT is mainly expressed on the apical membrane of enterocytes in the terminal ileum, but it can also be found at lower levels in the kidneys, where it hypothetically prevents loss of BAs in urine, and in cholangiocytes to form a smaller cholangio-hepatic loop [19].

IBATis are small molecules not absorbed into systemic circulation that bind IBAT in the distal ileum, preventing reabsorption and causing massive fecal loss of BAs. Historically, this class of drugs was developed as cholesterol-lowering agents, as the loss of BAs activates hepatic production via the farnesoid X receptor and consequently the consumption of cholesterol as a precursor of the BA synthetic pathway [20].

The availability of more effective drugs with less side effects for the treatment of hypercholesterolemia led to the evaluation of IBATis as a cure for cholestatic diseases, cholestatic pruritus and even constipation [21]. Indeed, the major side effect of IBATis is the softening of stools or mild diarrhea due to increased motility secondary to the massive passing of BA in the colon. This effect, however, is generally transient.

To date, five molecules have been developed but only two of them have been studied in children. Odevixibat was the first to complete registration trials for PFIC [22] while maralixibat first completed trials for ALGS [23]. However, they have been licensed in the US and Europe for both diseases now. It is worth noting that in mouse models the reduction in BA concentration was greatest in the blood compartment, but a 65% reduction was also observed intrahepatically [24]. Improvement of inflammation and fibrosis was also observed, likely due to the reduction in BA-mediated injury [25].

On the other hand, the efficacy of IBATis is critically dependent on the ability of BAs to be transported in the intestine and consequently lost. Indeed, the effect of reducing blood concentrations is maximal in healthy subjects and virtually nil in severe obstructive cholestasis. This limitation has significant implications for the treatment of genetic cholestasis in children.

## 5. Progressive Familiar Intrahepatic Cholestasis

### 5.1. Natural History

Over the last decade, knowledge on PFIC has grown thanks to the NAPPED (Natural Course and Prognosis of PFIC and Effect of Biliary Diversion) consortium [26]. The NAPPED registry collected the retrospective data of more than 800 patients, mainly with PFIC1 and PFIC2, from >70 centers from all continents. Despite the heterogenicity of the data, a few major issues such as natural history, genotype–phenotype correlation and response to surgical biliary diversion (SBD) have been addressed.

PFIC1 patients showed a native liver survival (NLS) of 70%, 59% and 44% at, respectively, 5, 10 and 18 years of age. SBD was commonly performed in these patients; 62 out of 130 patients (48%) underwent SBD at a median age of 5,9 years, and 15 of them (24%) later received liver transplantation (LT) with a median time from SBD of 2,5 years. The benefit of SBD has only been suggested for a minority of patients who respond with a marked decrease in sBA. The NLS cumulative curve was similar between children who did or did not receive an SBD, but the cut-off of 65 μmol/L for sBA after SBD was able to identify a subgroup of patients with better outcome. A sustained relief of pruritus, however, was noted only in a fraction of those patients [27].

As regards biomarkers of severity, only degree of cholestasis at disease onset, as reflected by sBA and serum total bilirubin (TB) concentrations under the cut-off value of 191 μmol/L and 72 μmol/L, respectively, was associated with better long-term NLS.

Indeed, no genotype–phenotype correlation was found. The presence of one or even two truncating mutations was not associated with a worse NLS or SBD outcome [27].

A larger cohort of 264 patients with PFIC2 was studied within the NAPPED consortium.

PFIC2 was confirmed to be a severe disease, 16 patients died during a median follow-up time of 4,1 years and only 32% of them were alive with their native liver at the age of 18 [28].

Severity of disease was easily predictable according to genotype. NLS at 18 years was 55% in patients with at least one of the two milder known missense mutations, p.E297G or p.D482G, less than 30% in patients with at least another missense mutation and virtually none of patients with two truncating mutations survived with their native liver [28]. A recent analysis found that the most negative prognostic factor is the presence of even a single truncating mutation, regardless of the second mutation [29]. Once more, sBAs have been identified as predictors of outcome after SBD. A decrease of at least 75% in sBAs or a level <102 μmol/L after SBD was associated with improved NLS [28]. These findings influenced the design of registration trials of IBATis as discussed below.

PFIC3 is the most common high-GGT variant. A wide spectrum of phenotypes in children and adults is linked to *ABCB4* deficiency [10]; pediatric onset diseases are those characterized by more severe genotypes, usually biallelic mutations [30]. The clinical picture is characterized by high-GGT cholestasis with progressive fibrosis and portal hypertension. Histology reveals ductular cholestasis, inflammatory cholangitis and various degrees of fibrosis. PFIC3 is a severe disease, the NAPPED consortium has not yet collected enough cases to determine the natural course of the disease. However a long-term follow-up of a historical case series from the Bicêtre group in France has recently been published. In total, 71% of patients had cirrhosis at a median age of 8.1 years, and 66% of these patients received a liver transplant at a median age of 8.5 years. NLS strictly correlated with severity of genotype. Patients with two truncating mutations were all transplanted before 10 years of age, while over 60% of patients carrying at least one missense mutation were alive with native liver at 20 years of age, albeit with varying degrees of fibrosis [31].

Ursodeoxycholic acid (UDCA) has shown some efficacy as a treatment for PFIC3 [32]. It produces a more hydrophilic pool of BAs, which reduces the detergent and toxic power of the phospholipid-poor bile of these patients. Patients who have mutations resulting in a higher functional residue of MDR3 are more prone to respond to UDCA, and biliary phospholipid levels above 6.9% predicted response to UDCA therapy. Only UDCA responders were able to reach adult age with their native liver [31].

### 5.2. Registrational Trials of IBATis in PFIC

The pharmacologic strategy of IBAT inhibition with odevixibat to reduce sBAs and cholestatic pruritus was tested in a phase 1 study [33] and then validated in a phase 2 study conducted in Northern Europe [34] (Table 2). In this open-label trial, 20 patients with chronic cholestasis were treated with different doses of odevixibat (10–200 mcg/kg). Interestingly, in addition to 11 patients with PFIC, 6 patients with ALGS and 3 with biliary atresia were also enrolled. The PFIC patients were as follows: one with PFIC1, seven with PFIC2, two with PFIC3 and one patient with PFIC10. Changes in sBA from baseline were the primary efficacy endpoint. Mean change after 4 weeks of treatment was −165.1 mmol/L (range, 394 to 1.2) in patients with PFIC. All patients showed a reduction in sBA, except one patient who carried an intronic splice site mutation in *ABCB11* with complete absence of the BSEP protein. As for secondary endpoints, pruritus and sleep disturbance were significantly reduced in most patients [34].

From this experience, the doses of 40 and 120 mcg/kg were selected for the subsequent phase 3 study, called PEDFIC-1, a 24-week, randomized, double-blind trial of odevixibat versus a placebo that enrolled 62 patients, 17 with PFIC1 and 45 with PFIC2 (Table 2). Patients with two truncating mutations of *ABCB11* and likely a complete absence of functional BSEP were excluded. Primary endpoints were a reduction from the baseline of pruritus measured with a ObsRO PRUCISION instrument [35] and a ≥70% reduction in sBA or sBA ≤ 70 μmol/L. At week 24, 33% of treated patients achieved sBA endpoint vs. none in the placebo group, and 55% of treated patients vs. 30% in the placebo group reached the pruritus endpoint. PFIC2 patients experienced a better response than PFIC1 with 40% vs. 17% of the sBA outcomes reached [36]. PEDFIC-1 continued as PEDFIC-2, a 72-week extension, open-label study with all patients receiving 120 mcg/kg. The results were published in 2023 considering a data cut-off of 24 weeks in PEDFIC-2 with up to 48 weeks of cumulative odevixibat exposure [37]. Interestingly, sBA responders, based on the criteria of PEDFIC-1, increased from 33% at week 24 to 53% at week 48, suggesting that in some cases six months of treatment are not enough to measure final efficacy. Moreover, 61% of patients enrolled directly in PEDFIC-2 achieved the pruritus endpoint after 24 weeks of treatment. The presence of patients with PFIC other than type 1-2 probably influenced this result [37].

Stratified, long-term results were recently presented at international meetings. At the end of PEDFIC-2, a total of 36 patients with PFIC1 were treated: 2 patients interrupted treatment and 14 were pruritus responders (39%), but only 5 (14%) fulfilled the sBA reduction outcome. Response was sustained after up to 96 weeks of therapy [38] (abs). As for PFIC2, 26 out of 69 patients were sBA responders (37%) at week 24. sBA response was largely concordant with pruritus response. sBA responders had early and sustained reductions both in sBA and in pruritus [39] (abs). As regards safety, odevixibat was generally well tolerated, as expected for an unadsorbed molecule, and drug-related adverse events occurred in 29% of patients, all mild or moderate, with none of them leading to discontinuation of therapy. The most common were increased ALT (5.7%) and bilirubin (10%). Surprisingly, diarrhea was rarely reported, and no worsening fat-soluble vitamin deficiency refractory to supplementation was reported [36,37].

Secondary analysis, not published as a peer-reviewed paper, focused on medium term outcomes. All patients who responded, at least partially in terms of sBA and/or pruritus, were alive with their native liver after three years [40] (abs), and event-free survival was 90% compared to less than 50% of a matched cohort extracted from the retrospective NAPPED data [41] (abs).

Maralixibat has also recently been licensed both in Europe and the US for PFIC treatment.

The first trial of maralixibat for PFIC was the INDIGO study [42] (Table 1). It was an open-label, phase 2, long-term study that enrolled 33 patients, 8 PFIC1 and 25 PFIC2. Six PFIC2 patients carried biallelic, protein truncating mutations and 19 had at least 1 non-truncating mutation. Patients received maralixibat 266 μg/kg until week 72 and then eventually increased to 266 μg/kg twice daily. Long-term efficacy was assessed at week 240. Pruritus was assessed using the ItchRO(Obs) score [43]. sBA response (reduction in sBAs of >75% from baseline or concentrations < 102.0 μmol/L as suggested by the NAPPED) was achieved in only seven patients (21%), all with non-truncating mutations and with doses of 266 μg/kg once daily in six of them [42].

The trial that successfully led to approval in PFIC was published in 2024 [44]. The MARCH-PFIC study enrolled 93 patients in three cohorts: patients with biallelic, non-truncated BSEP deficiency, patients with PFIC other than PFIC2 and a placebo. The dose was 570 mcg/kg/die, and treatment duration was 26 weeks. The primary endpoint was the mean change in pruritus severity score, and the key secondary efficacy endpoint was the mean change in total serum bile acids. The mean decrease from baseline in the pruritus score was 1.8 points in treated patients vs. 0.6 in the placebo group, and the mean change in sBA was −160 μmol/L vs. an increase of +3 μmol/L in the placebo group. Globally, 63,6% of patients met the pruritus endpoint criteria vs. 25,8% in the placebo cohort, and 54% vs. 6,5% met the sBA endpoint. Patients with non-BSEP PFIC showed a better response in terms of pruritus [44]. Preliminary, non-peer-reviewed data from the MARCH-ON study, a single-arm extension protocol that was also comprised of patients from the MARCH-PFIC, showed that patients with PFIC1-2 who achieved the sBA endpoint showed a better 2-year outcome than those with an insufficient reduction in sBA [45] (abs). Also, growth and not surprisingly quality of life seemed to be positively affected by a reduction in pruritus [46,47] (abs).

### 5.3. Real-World Experience

Clinical trials are generally conducted in a very selected population, with strict inclusion and exclusion criteria. This type of study does not always reflect the complexity of different clinical situations, especially in rare diseases. Real-world studies are observational studies conducted after drugs are commercially available. They are a precious and selection-unbiased tool to assess the efficacy of new drugs under day-by-day conditions (Table 1). The Expanded Access Program launched in Italy in 2021 allowed clinicians to treat PFIC patients with odevixibat including children who could not be enrolled in trials. A total of 24 patients have been treated, including rarer forms of PFIC: 5 PFIC4, 1 PFIC5, 1 PFIC10 and 1 PFIC9. Baseline data depicted a quite cholestatic population with pretreatment sBAs of 317 μmol/L, severe pruritus in most patients and signs of portal hypertension in seven of them. Using PEDFIC-1 criteria, reduction in sBA was remarkably higher than in PEDFIC1-2: responders were 54% at 3 months and 75% at 6 months. Similarly, pruritus responders at 6 months were as much as 73%. Stratifying patients per PFIC types, patients with PFIC1-2 showed a lower response rate, 63% at 6 months for sBA, but still outperformed PEDFIC1-2. Six patients did not respond, five of which had PFIC2, including two patients with truncated protein and one with PFIC1 [48]. In the whole cohort, a reduction in sBA levels of 50% from baseline at 1 and 3 months predicted a full pruritus response at 6 months in a recent sub-analysis only available as an abstract [49] (abs).

Variants other than 1 and 2 showed an unexpected excellent response, with all patients meeting the sBA endpoint at 6 months. In particular, TJP2 deficiency (PFIC4) appears to be the PFIC variant that respond better to IBATis. In a subgroup of PFIC4 patients, improvement of pruritus was terrific, from very severe to none in one case, and all patients showed a reduction in sBA ≥ 70% from baseline [50]. These results are consistent with the mechanism of disease in PFIC4 (Figure 1B). In these patients, the functionality of membrane transporters was intact, and cholestasis results from reflux of bile through defective tight junctions. A great proportion of BAs can reach the ileus and be lost in stools (Figure 1D). Also, in PFIC10, odevixibat seems very effective. In a small European series of patients, by 6 months all patients achieved sBA levels < 10 µmol/L, and total bilirubin fell to <15 µmol/L [51]. As for safety, adverse events were more frequent in real-world studies than in RCT. In total, 17% and 13% of patients reported, respectively, diarrhea and hypertransaminasemia, but they were usually transient and mild [48].

A smaller case series from Germany included nine patients with PFIC1-2. In that instance, all patients showed a marked reduction in sBAs except in two with complete loss of bile salt export pump due to truncating mutations, and eight out of nine patients experienced improvement of pruritus [52]. In this paper, two patients with an episodic form of PFIC2, previously called benign recurrent intrahepatic cholestasis (BRIC), were treated, resulting in a slow but remarkable improvement of symptoms.

Another small series of six patients with episodic intrahepatic cholestasis secondary to biallelic *ATP8B1* mutations (BRIC1) was recently published. Treatment was able to improve acute episodes with significant relief of pruritus and quality of life but apparently could not prevent recurrences [53].

Patients with PFIC1 may develop post-transplant diseases, characterized by severe diarrhea, metabolic decompensation, steatosis and steatohepatitis leading in most cases to graft loss. Performing SBD at the same time as transplantation had already been suggested. Early odevixibat therapy can improve diarrhea and quality of life in these patients, making SBD obsolete [54,55].

Data from the multicenter prospective registry TREATFIC will be available soon. To date, this registry is rapidly expanding and currently includes 84 patients, and 42 of them were treated with IBATis (33 odevixibat, 9 maralixibat) [56] (abs).

**Table 2:** Published studies on PFIC patients.

Refs	Author	Alias	Type	Pts	Molecule	Dose (μg/kg)	Duration	Pruritus Endpoints	Results	sBA Endpoints	Results
[34]	Baumann, U. et al.	None	Ph.2 OLNR	15 ^a^	ODX	10–200	4 w	Reduction ^b^	11 responders	sBA reduction	14 responders −165.1 μmol/L
[36]	Thompson, R.J. et al.	PEDFIC1	Ph.3 RCT	62	ODX	40–120	24 w	PPA ^c^	55% vs. 30% placebo	Change in sBA sBA red. > 70% or sBA ≤ 70 μmol/L	−114.3 vs. +13.1 33%
[37]	Thompson, R.J. et al.	PEDFIC2	EXT	69 ^d^	ODX	40–120	24 or 48 w	PPA	33% treated 61% naive	Change in sBA sBA red. > 70% or sBA ≤ 70 μmol/L	−201 μmol/L 53% at 48 w
[48]	Di Giorgio, A. et al.	None	RW	24	ODX	40–120	3–6 m	PPA	63–73%	sBA red. > 70% or sBA ≤ 70 μmol/L	54–75%
[50]	Di Giorgio, A. et al.	None	RW	5	ODX	40	5 m	PPA	100%	sBA red. > 70% or sBA ≤ 70 μmol/L	100%
[51]	Roquelaure, B. et al.	None	RW	5	ODX	37.2–120	6 m	Reduction	4 out of 5	sBA < 10 μmol/L	4 out of 5
[52]	Marx, M. et al.	None	RW	9	ODX	40–130	5–11 m	Reduction	7 out of 9	sBA reduction	7 out of 9
[53]	Di Giorgio, A. et al.	None	RW	6	ODX	28–120	NA ^e^	Reduction	Marked 66% Partial 33%	sBA reduction	3 out of 6
[55]	Vogel, G.F. et al.	None	RW	9	ODX	30–120	8–22 m	NA ^f^	NA ^f^	NA ^f^	NA ^f^
[42]	Loomes, K.M. et al.	INDIGO	Ph.2 OLNR	33	MRX	266	72–240 w	Reduction ^g^	10 responders	sBA red. > 75% or sBA < 102 μmol/L	7 responders ^h^
[44]	Miethke, A.G. et al.	MARCH-PFIC	Ph.3 RCT	93	MRX	142.5–1140	26 w	Mean change ^i^ Response ^l^	−1.8 vs. 0.6 placebo 64%	Mean change sBA red. > 75% or sBA < 102 μmol/L	−160 vs. +3 μmol/L 50%

**Abbreviations.** Pts: patients; OLNR: open-label non-randomized; RCT: randomized controlled trial; EXT: open-label extension trial; RW: real-world observational study; sBAs: serum bile acids; NA: not applicable; red.: reduction; w: weeks; m: months; Ph.: phase. **Notes.** ^a^ Four patients enrolled two times at different doses. ^b^ Multiscore (VAS, Whitington, PO-SCORAD). ^c^ Positive pruritus assessment: reduction in pruritus > 1 point with ObsRO PRUCISION [35]. ^d^ Including 53 patients enrolled in PEDFIC1 [36]. ^e^ Not applicable for heterogeneity of treatment schedule. ^f^ Indication to treatment: diarrhea and steatosis. ^g^ Clinically significant reduction: >1 point Itch(RO) score [43]. ^h^ All with non-truncating BSEP mutations. ^i^ Itch(RO) score [43]. ^l^ Average morning ItchRO(Obs) severity score reduction from baseline to the last 12 weeks of treatment (weeks 15–26) of at least 1 point or an average severity score of 1.0 or less over the same time period.

## 6. Alagille Syndrome

### 6.1. Natural History

ALGS is a systemic disease. There is significant long-term mortality, more often due to extrahepatic disease. NLS was estimated in historical cohorts between 50 and 65% at age of 10 years and 25–60% at the age of 20. Among patients who died with their native liver, liver-related disease was the cause in only one-third of cases [57]. The most frequent causes of death are cardiac or vascular/hemorrhagic episodes [58]. Cardiovascular phenotype is the most important determinant of outcome. Patients with structural heart disease suffered from up to 60% mortality before 10 years of age in the largest series [59,60]. Liver disease, however, is the primary cause of morbidity due to chronic courses and a frequent need for LT. A cholestatic picture characterizes the first years of age, and patients with neonatal cholestasis have a faster disease progression and earlier need for transplant. At this stage, jaundice, pruritus, xanthomas and failure to thrive are the most common symptoms. In patients surviving with their native liver, cholestasis decreases with age, and the clinical picture slowly evolves towards signs of portal hypertension [59]. A threshold for total bilirubin (TB) of 3.8 mg/dL between 12 and 24 months was identified as a risk for poor outcome in a multicentric study in the UK and North America [61].

As in the case of PFIC, another international consortium, the Global Alagille Alliance (GALA) Study Group, collected data from more than 1500 patients from 34 countries around the world [62]. The results refined several existing concepts. Cumulative NLS was consistent with previous studies, but the size of the population allowed early predictors of poor outcome to be better predicted. Bilirubin was the most important, and children > 6 and ≤12 months of age with median TB levels of ≥10.0 mg/dL have an increased risk of LT, with a hazard ratio (HR) of 15.6 and an 8.0-fold HR to develop clinically evident portal hypertension versus patients with TB < 5.0 mg/dL.

Overall, 72% of transplants were performed before 5 years of age. The most common indication (70%) was cholestasis and pruritus at a median age of 2.5 years. The remaining 30% of transplants were performed for decompensated portal hypertension at a median age of 4.2 years [62].

In a secondary analysis, not fully published, sBAs were also a prognostic factor for NLS, as expected, and remained significant, adjusting for TB clearance at 1 year. A median sBA in the first 3 years above 102 μmol/L predicted lower NLS at 8 years of age [63] (abs).

### 6.2. Registrational Trials of IBATis in ALGS

Studies reporting the results of treatment with IBATis in ALGS are summarized in Table 3.

The ICONIC study was the main RCT for maralixibat in ALGS. Published in 2021, it was a placebo-controlled, randomized withdrawal period (RWD), phase 2b study with open-label extension. After 18 weeks of maralixibat dosed at 380 μg/kg once per day, patients were randomized to continue maralixibat or receive a placebo for 4 weeks and then open-label maralixibat until week 48. Thereafter, during long-term extension, the study doses were increased up to 380 μg/kg twice per day. The primary endpoint was the mean sBA changing after withdrawal in participants with at least 50% sBA reduction by week 18. This complex trial design was specifically designed to minimize the placebo effect [64]. In total, 15 out of 29 patients (51.7%) achieved an sBA reduction of at least 50% from baseline to week 18. At the end of the RWD, mean difference in sBA between the maralixibat and placebo groups was −114 µmol/L. During the core 48-week period, in the overall population, 24 (83%) of 29 participants had an sBA reduction of at least 20%. Reduction in sBAs from baseline to week 48 was −96 µmol/L, and pruritus improved significantly with a −1.6 ItchRO(Obs) score. Maralixibat was well tolerated, and most adverse events occurred in the first weeks and were self-limiting. Diarrhea and abdominal pain were the most frequent [64].

In 2023, data from 76 patients enrolled in ICONIC and in the earlier ITCH (NCT02057692) [65] and IMAGO (NCT01903460) studies were aggregated. The average duration of treatment with maralixibat was 4.7 years, and the aim of this analysis was to identify predictors of EFS and NLS. Overall, EFS and NLS at 6 years from the start of maralixibat were 76% and 79%. TB at week 48 was the strongest predictor of EFS with an optimal threshold of 6.5 mg/dL (94% <6.5 mg/dL vs. 42% >6.5 mg/dL). Reductions of >1 point of ItchRO(Obs) pruritus score and sBA < 200 µmol/L at 48 week were also good predictors of EFS. Patients with three negative criteria had 33% EFS at 6 years vs. 97% of those with three positive predictors [66]. Durability of response and safety was confirmed in the same cohort by the MERGE study for up to 7 years of treatment [67] (abs).

A further secondary analysis was conducted comparing children treated with maralixibat in all trials with a matched historical series extracted from the GALA cohort. Six years of EFS was significantly better in treated patients (71.4%) than controls (50%) [68].

In 2022, a combined analysis of the extension studies IMAGINE I and II was published. At week 48, a significant reduction of −1.6 points of ItchRO(Obs) score and a reduction of −79.9 µmol/L was observed. Decrease was durable at week 72 and at end of treatment. In total, 34 of the 45 participants at stable dosing after week 48 were receiving 280 mcg/kg/day [69] (Table 3).

The RCT of odevixibat in ALGS was completed later and published in 2024. The ASSERT study (NCT04674761) was a phase 3, double-blind, randomized, placebo-controlled trial involving 69 patients. The primary efficacy endpoint was a change in pruritus score with the PRUCISION instrument [35] from baseline to weeks 21–24. The secondary efficacy endpoint was a change in sBA from baseline to the average of weeks 20 and 24. Itching improved by 1.7 points in treated patients vs. 0.8 in the placebo group, sBAs decreased by 90 µmol/L vs. an increase of 22 µmol/L in the placebo cohort. The most common adverse event was diarrhea (29% in the odevixibat group vs. 6% in the placebo group). Two treated patients had ALT elevations of at least three times higher than baseline, one interrupted odevixibat for 40 days and returned to baseline, and in the other ALT improved without interruption [70].

**Table 3:** Published studies on Alagille patients.

Refs	Author	Alias	Type	Pts	Molecule	Dose (μg/kg)	Duration	Pruritus Endpoints	Results	sBA Endpoints	Results
[65]	Shneider, B.L. et al.	ITCH	Ph.2b RCT	37	MRX	70, 140, or 280	13 w	Mean change in ItchRO(Obs) score	Not met	Mean change in sBA	No significant reduction in sBA
[64]	Gonzales, E. et al.	ICONIC	Ph.2b RWD	31	MRX	380–760	18 + 4 w ^a^ 48 w ^b^	Mean difference in ItchRO(Obs) score during RWD	−1.5 points −1.6	≥50% sBA reduction by Week 18 Mean sBA difference during RWD Mean change from baseline	51.7% −114 µmol/L −96 µmol/L
[69]	Shneider, B.L. et al.	IMAGINE I/II	EXT	53	MRX	140–560	48 w	Mean change in ItchRO(Obs) score > One point reduction	−1.59 73%	Mean change in sBA	−79.9 µmol/L
[70]	Ovchinsky, N. et al.	ASSERT	Ph.3 RCT	52	ODX	120	24 w	Mean change in scratching score (PRUCISION)	−1.7 (ODX) −0.8 (Placebo)	Mean change in sBA	−90 µmol/L (ODX) +22 µmol/L (Placebo);
[71]	Himes, R. et al.	None	RW	8	MRX	380	12–28 w	Reductions in CSS ^c^	All patients	NA	NA

**Abbreviations.** Pts: patients; RCT: randomized controlled trial; RWD: randomized withdrawal period; EXT: open-label extension study; RW: real-world observational study; sBA: serum bile acids; NA: not applicable; w: weeks; m: months; Ph.: phase. **Notes.** ^a^ Four weeks of randomized withdrawal after 18 weeks of treatment. ^b^ Open-label extension. ^c^ Clinician scratching score.

### 6.3. Real-World Experience

At the time of writing this review, there is only one published article containing real-world data (Table 2). It concerns a small group of eight North American patients excluded from trials because of previous SBD, reduction in concomitant medications, administration via gastrostomy or nasogastric tube or being under consideration for LT [71].

Other data come only from conference proceedings and must be considered with caution. In Europe, two real-world cohorts are being studied, and the preliminary results were recently presented. To date, the French cohort includes 46 children, with a median age of 3.6 years. They have been treated for a median duration of 12 months, including eight patients who interrupted treatment early. In total, 38/46 patients (82%) are defined as clinical responders and 29/46 (63%) as biological responders, while 26 of them experienced both an improvement in pruritus and a decrease in sBA; diarrhea and/or abdominal pain were reported in 52% of patients and in nine were the cause of treatment withdrawal [72] (abs).

The Italian Marea study (maralixibat expanded access for Alagille syndrome) is a real-world study that enrolled 13 patients. Median sBA decreased from 155 μmol/L to 51 μmol/L after 24 weeks of treatment and the ItchRO score from 2 to 1. In total, 77% of patients had a reduction in sBA of more than 25%. No significant changes in AST/ALT activity, TB and total cholesterol were observed at 3 and 6 months of treatment. To date, only very preliminary results have been presented [73] (abs).

As for safety, among the 37 patients enrolled in the Expanded Access Program in the US, only three patients (8%) experienced gastrointestinal treatment-related mild adverse events, two patients required dose reduction for diarrhea and one patient interrupted dosing for mild liver enzyme elevation. Only one grade 3 event was observed, and one patient had elevated liver enzymes that led to the only case of maralixibat discontinuation (3%). No patient experienced fat-soluble vitamin deficiency [74] (abs).

In European studies, experiences of diarrhea and/or abdominal pain were commonly observed (33–52%) but only occasionally led to treatment discontinuation (9 out of 54 patients in the French cohort) [72] (abs).

Indirect proof of efficacy comes from data on concomitant medications in patients who have joined the Mirum Access Plus program in the US. During maralixibat treatment, 43% of patients discontinued use of more than one concomitant medication, 44% discontinued a pregnane X receptor (PXR) agonist as rifampicin and 12% discontinued UDCA. Interestingly, 22% of children treated discontinued a nutritional or vitamin supplement [75] (abs). Long-term safety studies are currently ongoing both in Europe and in the US (NCT04168385, NCT06193928).

## 7. IBATis in Biliary Atresia and Other Cholestatic Diseases

### 7.1. Biliary Atresia

Kasai portoenterostomy remains the primary treatment aimed at restoring bile drainage; however, even when successful, many patients experience ongoing cholestasis and require liver transplantation during childhood or adolescence. Accumulation of BAs within the liver is a key driver of inflammation, fibrosis and hepatocellular damage in biliary atresia [76,77,78]. Moreover, chronic cholestasis contributes to significant morbidity, including refractory pruritus, fat-soluble vitamin deficiency and growth failure. These pathophysiological features suggest that interrupting the enterohepatic circulation may reduce BA-mediated injury and possibly improve clinical outcomes in patients with biliary atresia [79]. In the already-mentioned phase 2, open-label, multicenter study, three patients were treated with odevixibat with daily oral doses ranging from 10 to 200 µg/kg over a four-week period. Two out of three exhibited approximately 50% reductions in serum bile acid levels. The remaining patient, who had a comparatively low baseline sBA (42.8 µmol/L), did not experience a similar decline. Additionally, two of the three patients in this subgroup also showed a reduction in pruritus [34].

In 2025, Nicastro et al. described a series of seven patients with biliary atresia and residual cholestasis after a Kasai procedure treated with odevixibat (40 µg/kg QD) [80]. All the children had a complete response to pruritus after 2 weeks with a median decrease in serum bile acids of −75% (55–86%) compared to baseline values (239 ± 115 μmol/L). The treatment was safe with transient mild diarrhea recorded in only one patient. Currently, two clinical trials are registered on ClinicalTrials.gov investigating the potential role of odevixibat. NCT04336722 (BOLD study) is a double-blind, randomized, placebo-controlled, phase 3 study investigating the efficacy and safety of odevixibat compared to a placebo in children with biliary atresia who have undergone a Kasai procedure. NCT05426733 (BOLD-EXT) is the extension study of the BOLD study with a treatment period of 104 weeks.

On the other hand, the EMBARK study (NCT04524390) evaluating maralixibat in biliary atresia response post-Kasai was completed, and the results are available online [81]. The study population consisted of children aged up to 110 days within 3 weeks after Kasai surgery. The primary outcome of a reduction in TB was not achieved. Among the secondary outcomes, only a reduction in sBA was significant while none of the measures reflecting the progression of diseases was different in the treated group. Interestingly, a reduction in pruritus was not included as an outcome.

Biliary atresia is a severe and quickly progressing disease, and it is likely that only older children with the partial benefit of a Kasai procedure and chronic pruritus may benefit from IBATis treatment.

### 7.2. Other IBATis Treatment Experiences in the Pediatric Population

Owing to their ability to reduce BA levels and pruritus, individual case reports have documented the use of IBATis in atypical clinical scenarios distinct from ALGS and PFIC. Goel et al. described a series of pediatric patients with rare variants of genetic cholestatic liver disease treated with odevixibat [82]. One of the patients was a 7-year-old boy with a history of idiopathic neonatal hepatitis presented with cholestasis and severe pruritus. Genetic testing showed a heterozygous variant in *AKR1D1* and homozygosity for a common *ABCB11* polymorphism. Liver biopsies over time revealed progression to sclerosing cholangitis, and he was later diagnosed with inflammatory bowel disease. Standard antipruritic treatments were insufficient, and odevixibat was introduced resulting in a marked reduction in sBA, improved pruritus, better sleep and enhanced quality of life, with no adverse effects reported. A 21-month-old girl with persistent cholestasis and pruritus was found to have progressive liver disease with features of sclerosing cholangitis with intrahepatic bile duct abnormalities, despite normal extrahepatic bile ducts. Genetic testing revealed variants of uncertain significance in *PKHD1* and *PKHD2*, with no definitive molecular diagnosis. She was started on odevixibat, which only led to a transient decrease in sBA and mild improvement in pruritus.

Ganschow et al. reported the case of a 2-year-old boy with hypoplastic left heart syndrome and Kleefstra syndrome who developed severe cholestatic pruritus unresponsive to standard therapies. Off-label treatment with odevixibat (40 µg/kg/day) led to a rapid and sustained improvement in pruritus, sleep and quality of life, along with a significant reduction in serum bile acids (from 111 to 24 μmol/L after 1 month of treatment) [83]. Our group reported the case of a 13-year-old boy who developed vanishing bile ducts syndrome as a consequence of drug-induced liver injury in the setting of severe cutaneous adverse reactions with eosinophilia and systemic symptoms (DRESS) after starting treatment with ethosuximide. The patient experienced severe cholestasis and pruritus refractory to conventional treatments, and initiation of odevixibat therapy led to clinical improvement with normalization of sBA and relief of pruritus [84]. Similarly, Kehler described the case of an adolescent male with vanishing bile duct syndrome secondary to Hodgkin’s lymphoma who benefited from treatment with odevixibat, which led to the resolution of pruritus and a significant reduction in sBA [85] (abs).

## 8. IBATis Treatment Experiences in Adults

### 8.1. Primary Biliary Cholangitis (PBC)

Primary biliary cholangitis (PBC) is a chronic, progressive autoimmune liver disease of adulthood characterized by the destruction of intrahepatic bile ducts, leading to cholestasis and, over time, fibrosis and cirrhosis. It primarily affects middle-aged women and often presents with fatigue, pruritus and elevated cholestatic liver enzymes. Despite available treatments like ursodeoxycholic acid, many patients continue to experience symptoms—particularly pruritus—that significantly impair quality of life [86]. In 2018, a pilot study investigated the potential role of odevixibat in nine patients with PBC, treated with two different doses (0.75 mg and 1.5 mg) for four weeks, with the option to escalate the dose to 3 mg after one week if tolerated. All the patients reported an improvement in pruritus measured through a standardized scale, but five out of nine patients discontinued the study prematurely due to gastrointestinal side effects, primarily abdominal pain and diarrhea [87]. In 2019, a randomized, double-blind, placebo-controlled trial (NCT01904058, CLARITY trial) evaluated the impact of maralixibat on pruritus in 66 adults with PBC treated for 13 weeks or a placebo (primary endpoint) assessed with the adult ItchRO score (ranging from 0 to 70). The study demonstrated an improvement in the symptoms in both the maralixibat (−26.5; 95% confidence interval [CI]: −31.8, −21.2) and placebo (−23.4; 95%CI: −30.3, −16.4) cohorts. Among those treated with maralixibat, fasting serum bile acids levels decreased (least squares mean changes: −14.2; 95%CI: −28.2, −0.2), whereas they increased in the placebo group (+10.1; CI95%: −8.7, 28.8) (secondary endpoint). The maralixibat group experienced a higher incidence of gastrointestinal adverse events (78.6% vs. 50%), although none of the patients needed a dose interruption or drug discontinuation [88]. The authors speculated about the potential influence of the placebo effect on pruritus and emphasized the need for a careful trial design when pruritus is a subjective primary endpoint. Linerixibat is an IBAT inhibitor in evaluation as a treatment for cholestatic pruritus in PBC. In early studies, including a phase 2a cross-over trial, 2 weeks of linerixibat led to significant reductions in itch severity compared with a placebo [89]. The larger phase 2b GLIMMER trial (147 patients with moderate-to-severe itching) confirmed a dose-dependent improvement in pruritus scores over 12 weeks [90]. Recently, preliminary data from a phase 3 trial (NCT04950127, GLISTEN trial), conducted on 238 PBC patients treated with linerixibat (40 mg) or a placebo, showed that patients on linerixibat had significantly improved itchiness compared to the placebo (primary endpoint). Linerixibat was well tolerated, with diarrhea and abdominal pain reported in 61% and 18% of patients [91] (abs). Volixibat, a different IBAT inhibitor under development, is also under investigation for patients with PBC, in a 28-week, randomized, placebo-controlled, phase 2b study (NCT05050136, VANTAGE trial). An interim analysis of 31 patients, randomized equally to receive volixibat at 20 mg, 80 mg or a placebo, twice daily, reported an improvement in ItchRO scores and serum bile acids in patients treated with IBATi compared to the placebo. In total, 70% of volixibat-treated patients achieved a ≥50% reduction in serum bile acids. Diarrhea was the most common side effect, though generally mild; only one patient discontinued due to this [92] (abs).

### 8.2. Other IBATis Experiences in the Adult Population

Given the lack of effective therapies and the burden of symptoms such as pruritus, there is growing interest in targeting bile acid metabolism using IBAT inhibitors as a novel therapeutic approach in PSC. An open-label, phase 2 study (NCT02061540, CAMEO study) aimed to evaluate the impact of a 14-week treatment with maralixibat (at daily doses up to 10 mg) in a cohort of adult patients with PSC. In the overall population, after the treatment, serum bile acid concentrations decreased by 16.7% (−14.8 µmol/L; CI95%: −27.2, −2.4) compared to baseline (38.9 µmol/L, 23.6–54.2) and by 40% in participants with baseline levels above normal. Similarly, a reduction in ItchRO sum scores was outlined. Adverse events were mild (40.7%) or moderate (22%) and mostly gastrointestinal. Only one serious event (cholangitis) was related to treatment [93]. Another phase 2 trial is evaluating volixibat in PSC patients (NCT04663308, VISTAS study) but no published data are yet available.

Beyond their approved indications and ongoing trials, IBAT inhibitors have also been used off label in adult patients with various cholestatic conditions, with anecdotal reports suggesting potential benefits in managing pruritus.

Rodriguez et al. described a 36-year-old woman with a history of benign recurrent intrahepatic cholestasis (BRIC) who experienced severe intrahepatic cholestasis of pregnancy (ICP) in two consecutive pregnancies. In her first pregnancy, standard treatments with ursodiol and rifampin were insufficient to control bile acid levels or pruritus. During her second pregnancy, maralixibat was introduced in gradually increasing doses up to 380 μg/kg twice a day with mild improvement in pruritus and serum bile acids maintained under the threshold of 100 µmol/L [94]. The patient tolerated maralixibat well, though she developed fat-soluble vitamin deficiencies and steatorrhea, highlighting the need for close nutritional monitoring. A clinical trial (NCT04718961, OHANA study) was designed to investigate the effect of volixibat in ICP, but it was discontinued due to difficulties in patient recruitment. In the past, volixibat was tested for efficacy in adults with non-alcoholic steatohepatitis (NASH). Despite showing biochemical evidence of target engagement (increased bile acid synthesis and reduced cholesterol), it did not improve liver fat content on histology [95]. The treatment was generally well tolerated, but the study was terminated early due to lack of clinical benefit.

The inhibition of the ileal bile acid transporter reduces the reabsorption of bile acids in the terminal ileum. This increase in bile acids reaching the colon stimulates secretion and motility, thereby improving stool frequency and consistency. Because of this dynamic, IBATi has also been studied for the management of chronic constipation. Following the results obtained from phase 2 and 3 studies [96,97], elobixibat received approval from the Japanese Pharmaceuticals and Medical Devices Agency (PMDA) on 19 January 2018, for the treatment of chronic constipation of adult patients.

## 9. Discussion

It has almost been ten years since the first phase 1 study on odevixibat was published. Since then, more than 800 patients worldwide have been treated with the commercially available drug odevixibat and around 1000 with maralixibat. More importantly, data from real-world observational prospective cohort studies are beginning to become available (Table 2 and Table 3). These cohorts are not subject to the limitations of clinical trials, which typically enroll patients according to criteria designed to facilitate the achievement of endpoints. However, it should be noted that multicentric observational studies suffer from a certain heterogeneity in the enrollment criteria, and in particular, the local decision-making process that led to the start of treatment is unknown. In addition, for some of these studies, only non-peer-reviewed communications are currently available [72,73,74,75] (abs). Nevertheless, we believe that critically assessing and combining these data can provide a realistic overview.

Pruritus is one of the most disabling symptoms in cholestatic liver disease. Despite years of investigation, its origin remains elusive. A number of potential players such as lysophosphatidic acid, resulting after hydrolysis of lysophosphatidylcholine operated by the enzyme autotaxin, have been studied without definitive conclusions [98].

BAs are able to activate all pruritogenic receptors but not in physiological conditions, with the only exception being the Takeda G-protein coupled receptor (TGR5) [3]. Interestingly, serum from patients with a deficit of sodium taurocholate co-transporting polypeptide (NTCP), who typically do not suffer from itching, is equally able to activate TGR5 [3]. The direct role of BA in causing pruritus has never been demonstrated. We learned from early IBATis trials that no statistically significant correlations exist between baseline sBA and pruritus, but after treatment the change in SBA and autotaxin levels accurately reflects the change in itching [34]. In summary, plasma levels of conjugated bile salts and their secondary derivatives correlate well with pruritus intensity in the single patient, but there is great inter-individual variability [3].

IBATis are drugs that ultimately lower the blood concentration of BAs in 40–50% of patients with PFIC or ALGS and at the same time significantly reduce pruritus in 50–60% of these patients [36,37,42,44,64,70]. Interestingly, both registrational trials and real-world studies have shown that some patients with sBA levels that are still high show a measurable improvement in pruritus, while the contrary is only exceptionally observed. In other words, experience with IBATis has shown that a reduction in sBA strongly correlates with a reduction in itching, but a reduction in pruritus is not always associated with decreased sBA.

Moreover, experience with surgical enterohepatic circulation interruption in PFIC has shown that the main limitation of IBAT inhibition is that BAs must reach the intestine in order to be eliminated [28,29]. The network of proteins responsible for transporting BAs out of the hepatocyte must ensure a residual function that provides a rate of elimination greater than the hepatocyte’s maximum synthetic capacity. Given these conditions, it will only be a matter of time, in some cases even more than six months, before the BA pool is depleted and pruritus might improve.

It has been proven that patients with BSEP deficiency who carry variants producing nonfunctional/truncated protein do not show any benefits from SBD [34,42,99]. Consequently, only patients with at least one missense mutation of BSEP were enrolled in later trials such as the PEDFIC-1 study. A proportion of them, however, were not responders [36]. Looking for predictors of IBATis response, composition analysis of sBA on a pretreatment serum sample of 41 patients with PFIC2 enrolled in PEDFIC-1 showed that the baseline concentration of cholic acid (CA) and chenodeoxycholic acid (CDCA) was higher in responders than in non-responders [100]. A higher pretreatment unconjugated sBA concentration in responders is likely due to the passage to the colon of a greater amount of conjugated BA that undergoes deconjugation by colonic microbiota, indirectly confirming that residual BSEP function is the critical prerequisite for treatment response to IBATis. Accordingly, PFIC2 patients with higher pretreatment bilirubin levels were more likely non-responders after IBATis treatment. Similar results were found also after 24 weeks of treatment. The fraction of unconjugated sBAs were more than 70-fold higher, and the absolute concentration was 2-fold higher in responders than in non-responders and the placebo [97]. From a clinical point of view, missense BSEP mutations with a residual function are associated to milder phenotypes [101,102].

Relevance of unconjugated sBA as a marker of treatment response might also be confirmed in patients treated with maralixibat in the MARCH/MARCH-ON trials. Serum concentrations of CA and CDCA seem positively associated with a reduction in pruritus in patients with all PFIC variants [103] (abs).

Depletion of sBA in responders also causes a reduction in the production of fibroblast growth factor 19 (FGF19) by enterocytes and, consequently, an increase in hepatic synthesis due to the removal of negative feedback mediated mainly by farnesoid X receptor (FXR) stimulation. Treated patients have higher 7alpha-hydroxy-4-cholesten-3-one (C4) levels, a surrogate marker of hepatic BA synthesis rate [103] (abs). Higher C4 concentrations were found also in PFIC2 responders in the PEDFIC-1 study, but the increase during IBATis therapy, although significant, led to values comparable to healthy subjects [36]. In the INDIGO study, the C4 pretreatment level was higher in patients with truncating BSEP mutation and gradually increased only in responders during treatment with maralixibat [42].

From real-life experience, we observed that a treatment duration up to one year may be necessary to appreciate clinical results and that non-1–2 PFIC shows better outcomes [48].

Almost all patients who experience a clinical benefit show, in the long term, a highly variable decrease in sBA concentration, but the magnitude of this reduction does not parallel the improvement in pruritus often being less pronounced.

Whether this reduction in sBA will lead to a real change in the long-term natural history of the underlying disease is a much-debated topic. It will probably be necessary to wait many years to obtain the answer.

Studies that have attempted to compare treated patients with historical cohorts [41,68] (abs), using complex statistical methods, are not fully reliable for several reasons. Firstly, the historical cohorts themselves are affected by an intrinsic bias resulting from the extreme heterogeneity of management, particularly with regard to availability and the propensity for transplantation. Secondly, since pruritus is the main indication for transplantation in the early stages of the disease, particularly for ALGS, a significant reduction in the likelihood of transplantation is widely expected, due to the marked efficacy in reducing the symptom and also to the hope that new and even more effective treatments may emerge in the near future. What seems plausible is that patients with milder phenotypes, i.e., those who are less cholestatic, are the best candidates to obtain from BA depletion some kind of benefit in terms of the progression of fibrosis due to chronic cholestasis.

In terms of safety, it is now well established that IBATis are safe drugs. Diarrhea and abdominal pain cannot be considered true adverse events, but they are consequential and, in fact, directly related to the effectiveness of the IBAT block and the passage of BAs into the colon. Therefore, tolerance of these symptoms will be higher due to the greater relief from itching. This could explain the wide variability in reported frequency and the relatively higher incidence in real-life studies, which more often involved patients with milder phenotypes. The only notable true side effect is the occasional increase in transaminases, which has resulted in a small number of treatment interruptions. While this is usually only temporary, caution is still necessary, as the long-term consequences of this phenomenon are still unknown. Careful monitoring of α-fetoprotein levels appears reasonable. Contrary to expectations, it is clear that there is no real risk of fat-soluble vitamin depletion, given that most patients are already receiving supplementation before treatment onset [104] (abs).

The first guidelines incorporating the use of IBATis in the treatment of cholestatic pruritus have recently been published. They were developed by an EASL working group that included a significant number of pediatricians [105]. For pediatric patients with PFICs or ALGS, IBATis are recommended as a second-line treatment after rifampicin and UDCA have been tried, even though UDCA cannot be considered a drug solely for treating pruritus. On the basis of the data published to date, this position appears reasonable, but some further considerations can be made. Firstly, IBATis are effective in reducing sBA concentrations not only in PFIC and ALGS and might also be a valuable tool in selected patients with other obstructive conditions such as biliary atresia or sclerosing cholangitis. However, if the indication is limited to pruritus, the window of opportunity under such conditions, where pruritus is a late symptom of advanced disease, would likely be missed. As mentioned above, PFICs or ALGS patients who benefit most from this treatment are those who show moderate cholestasis with some residual ability to transport BAs in bile. In these patients, especially in conditions with biliary pathogenesis such as ALGS or MDR deficiency, pruritus may respond to rifampicin in the early stages and become refractory as fibrosis and obstruction progress. In these situations, it might be reasonable to consider IBATis as the first line of treatment, and this will become even easier with the reduction in treatment costs that is likely to occur in the coming years.

Finally, there are reports on the effectiveness of IBATis even in conditions that are not necessarily chronic, such as cholestatic drug-induced liver injury [84]. The EXPAND study (NCT06553768) is currently ongoing to evaluate the efficacy of maralixibat in the treatment of cholestatic pruritus under different pathological conditions.

## 10. Conclusions

Based on the data published to date (Table 2 and Table 3), we can reasonably conclude that IBATis are now a well-established treatment for cholestatic pruritus. Even though IBATis are currently labelled only for PFIC and ALGS, it is highly desirable that in the future IBATis will become part of the pediatric hepatologist’s toolbox for the treatment of cholestasis in all situations that may benefit from a significant reduction in sBA.

## Figures and Tables

**Table 1:** List of currently recognized PFIC variants.

	Gene	Protein	GGT
PFIC1	*ATP8B1*	FIC1	L
PFIC2	*ABCB11*	BSEP	L
PFIC3	*ABCB4*	MDR3	H
PFIC4	*TJP2*	TJP2	L
PFIC5	*NR1H4*	FXR	L
PFIC6	*SLC51A*	OSTα	H
PFIC7	*USP53*	USP53	L
PFIC8	*KIF12*	KIF12	H
PFIC9	*ZFYVE19*	ZFYVE19	H
PFIC10	*MYO5B*	MYO5B	L
PFIC11	*SEMA7A*	SEMA7A	L
PFIC12	*VPS33B*	VPS33B	L
PFIC13	*PSKH1*	PSKH1	H

PFIC: progressive familial intrahepatic cholestasis; GGT: gamma glutamyl transpeptidase; L: low; H: high.

## Data Availability

No new data were created or analyzed in this study. Data sharing is not applicable to this article.

## Abbreviations

IBAT: ileal bile acid transporter; IBATis: ileal bile acid transporter inhibitors; PFIC: progressive familial intrahepatic cholestasis; ALGS: Alagille syndrome; BA: bile acids; sBA: serum bile acids; LT: liver transplantation; RCT: randomized controlled trial; FIC1: familial intrahepatic cholestasis 1; BSEP: bile salt export pump; MDR3: multidrug resistance 3; GGT: gamma glutamyl transpeptidase; TJP2: tight junction protein 2; MYO5B: myosin-Vb protein; NOTCH: neurogenic locus Notch homolog protein; SBD: surgical biliary diversion; NLS: native liver survival; EFS: event-free survival; UDCA: ursodeoxycholic acid; ObsRO: observer-reported outcome; ALT: alanine aminotransferase; TB: total bilirubin; HR: hazard ratio; RWD: randomized withdrawal period; CA: cholic acid; CDCA: chenodeoxycholic acid; AASLD: American Association for the Study of Liver Diseases; EASL: European Association for the Study of the Liver; ESPGHAN: European Society for Pediatric Gastroenterology, Hepatology and Nutrition.
